# Current Studies of Acupuncture in Cancer-Induced Bone Pain Animal Models

**DOI:** 10.1155/2014/191347

**Published:** 2014-10-14

**Authors:** Hee Kyoung Ryu, Yong-Hyeon Baek, Yeon-Cheol Park, Byung-Kwan Seo

**Affiliations:** Department of Acupuncture & Moxibustion, Kyung Hee University Hospital at Gangdong, 149 Sangil-dong, Gangdong-gu, Seoul 134-727, Republic of Korea

## Abstract

Acupuncture is generally accepted as a safe and harmless treatment option for alleviating pain. To explore the pain mechanism, numerous animal models have been developed to simulate specific human pain conditions, including cancer-induced bone pain (CIBP). In this study, we analyzed the current research methodology of acupuncture for the treatment of CIBP. We electronically searched the PubMed database for animal studies published from 2000 onward using these search terms: (bone cancer OR cancer) AND (pain OR analgesia) AND (acupuncture OR pharmacopuncture OR bee venom). We selected articles that described cancer pain in animal models. We analyzed the methods used to induce cancer pain and the outcome measures used to assess the effects of acupuncture on CIBP in animal models. We reviewed articles that met our inclusion criteria. Injection of mammary cancer cells into the cavity of the tibia was the most frequently used method for inducing CIBP in the animal models. Among the eight selected studies, five studies demonstrated the effects of electroacupuncture on CIBP. The effects of acupuncture were assessed by measuring pain-related behavior. Future researches will be needed to ascertain the effectiveness of acupuncture for treating CIBP and to explore the specific mechanism of CIBP in animal models.

## 1. Introduction

Bone pain is a common type of cancer pain [1]. Bone metastasis can occur in advanced cancer, most commonly in breast, prostate, and lung cancer. Bone metastasis can occur in up to 70 percent of patients with advanced breast or prostate cancer and in approximately 40 percent of patients with lung, kidney, or thyroid cancer [2]. Although the exact rate of bone metastasis has not been determined, it was reported that over 400,000 individuals are affected in the United States annually [3].

Tumor growth in bone results in pain, hypercalcemia, anemia, increased risk of infection, pathological fractures, compression of the spinal cord, spinal instability, and decreased mobility. These factors threaten the patient's functional status, quality of life, and survival [2, 4]. Cancer-induced bone pain (CIBP) is a common symptom in cancer patients, and spontaneous breakthrough pain is severe and difficult to control [1].

The standard management of metastatic CIBP is a combination of radiotherapy and analgesic therapeutics. However, these interventions can fail to provide an adequate analgesic effect [5] and may have side effects. Side effects such as nausea, vomiting, diarrhea, and myelosuppression can occur due to radiotherapy, and constipation, nausea, drowsiness, and cognitive impairment are commonly associated with opioid-based analgesics [6]. Other medications such as calcitonin, bisphosphonate, denosumab, and corticosteroids are also prescribed to alleviate CIBP [7].

Acupuncture may represent a potentially valuable adjunctive strategy for pain alleviation, and it is known to be relatively free of harmful side effects [8]. Guidelines for acupuncture treatment for cancer patients state that acupuncture is effective for postchemotherapy symptoms such as nausea, vomiting, and xerostomia [9]. Additionally, acupuncture has been reported to be helpful for the relief of cancer pain [10, 11]. The clinical effects of acupuncture on CIBP have not been fully elucidated through rigorous randomized controlled trials. Animal models are essential for exploring and understanding the mechanisms of pain and developing effective therapies for its management [12]. Several animal models have been developed and used to investigate CIBP [13, 14].

To analyze the current research methodology of acupuncture for the treatment of CIBP, we investigated methods used to induce cancer pain and outcome measures used to assess the effects of acupuncture on cancer pain in various animal models.

## 2. Methods

### 2.1. Search Strategies

We electronically searched the PubMed database using the terms (bone cancer OR cancer) AND (pain OR analgesia) AND (acupuncture OR pharmacopuncture OR bee venom). Our search was limited to animal studies published from 2000 onward in any language.

We selected articles that described studies of animal cancer pain models. Review articles based on the literature and studies of animal models for other types of disease pain, such as polycystic ovarian syndrome and osteoarthritis, were excluded. We also excluded studies that did not focus on cancer pain and did not use pain-related outcome measures. The full texts of articles meeting the inclusion criteria were obtained and read carefully.

### 2.2. Data Extraction

The study design data were extracted and classified using a predefined data extraction form that designated the animal used (sex, species, and strain), the CIBP model type (type of cancer cell and site of tumor injection), the intervention type, and the outcome measures (pain-related behavior, macroscopic features, and histological, and biochemical measurements). The data were extracted primarily by one author and were checked by the other authors.

## 3. Results

**Figure 1:**
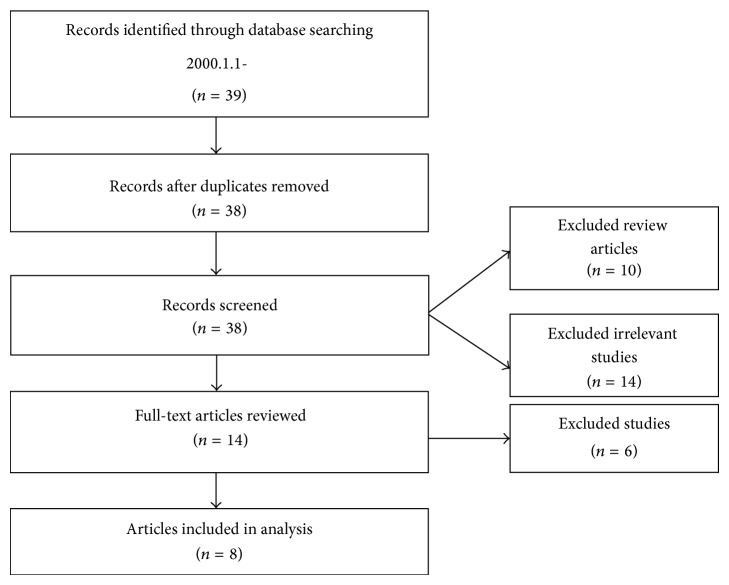
Flow diagram of the study selection process.

We retrieved 39 articles and read the titles and abstracts. There was one duplicate record. Of these 38 articles, 10 articles were reviews of the literature and 14 articles were disease studies that were not cancer-related (Figure 1). The remaining 14 articles were reviewed, and six were excluded because they did not focus on cancer pain. Finally, we selected eight animal model studies of CIBP for analysis.

We analyzed the animal models for CIBP in eight studies (Table 1). Five studies used Walker 256 mammary carcinoma cells to induce tumors, and seven studies involved injection of cancer cells into the tibial cavity. Five studies investigated the effects of electroacupuncture on CIBP (Table 2). We also analyzed the outcome measures used in the eight studies [15–22] (Table 3). Five studies assessed pain-related behavior that resulted from CIBP. Among these five studies, three assessed mechanical allodynia or hyperalgesia and four assessed thermal hyperalgesia. Four studies assessed indexes that increased with CIBP or cancer pain and decreased after electroacupuncture treatment [15, 18, 20–22].

## 4. Discussion

CIBP is a common symptom in cancer patients, and background pain, spontaneous pain, and movement-induced pain are the 3 main characteristics of CIBP. Background pain is a continuous dull ache that increases in intensity as time passes. Severe intermittent pain occurring spontaneously or upon movement or weight bearing is called breakthrough pain [23].

CIBP has a complicated relationship with pathological processes, neuroimmune mediators, tumor growth, and neuropathic conditions [24]. To explore these pain mechanisms, animal models have been developed to simulate specific human pain conditions in order to study the mechanisms and possible therapies [25].

Bone metastases have been classified as osteolytic or osteoblastic. In osteolytic metastases, the destruction of bone is mediated by osteoclasts. Patients with osteolytic metastases have severe pain, pathologic fractures, life-threatening hypercalcemia, spinal cord compression, and other nerve compression syndromes. Osteoblastic metastases can cause bone pain and pathological fractures because of the poor quality of the bone produced by the osteoblasts [26]. Osteolytic metastases occur in approximately 80 percent of patients with stage IV advanced breast cancer, and osteoblastic metastases occur in about 91 percent of patients with advanced prostate cancer [27]. The osteoclastic or osteoblastic pathway is not absolutely dependent on the tumor type because the interaction between osteoclasts and osteoblasts is complex [28]. However, it is clear that the osteoclast is the main factor responsible for bone metastasis pathology [4].

Our results revealed that injection of Walker 256 mammary carcinoma cells into the cavity of the tibia was the most frequently used CIBP animal model [15–19]. Earlier CIBP animal models were based on systemic injection of carcinoma cells. Disseminated malignancy, resulting in more than one randomly sited bone metastasis, occurred with the systemic injection model. In 1999, Schwei et al. [29] described the local injection of osteosarcoma cells into a single bone. At 21 days after the injection of osteolytic sarcoma cells into the cavity of the femur, extensive bone destruction and invasion of the tumor into the periosteum were observed, and these findings were similar to the pathological changes found in patients with osteolytic bone metastases. Thereafter, methods were developed in which breast cancer cells were injected into the tibia of rats, and fibrosarcoma, melanoma [30, 31], or adenocarcinoma [31] cells were injected into the humerus or femur of mice. Animals in these models exhibited clinical signs of bone metastasis such as bone destruction, limping, guarding, spontaneous flinching, decreased mobility, secondary hyperalgesia, and allodynia [30, 32]. These methods produced manifestations similar to human bone cancer symptoms but only partially reflected the pathological process of bone metastasis.

Behavioral tests are used to quantify pain in animals because animals can not directly express their experience of pain. The most commonly used tests are the radiant heat paw-withdrawal test and the von Frey test. The radiant heat paw-withdrawal test is used to assess thermal hyperalgesia. In this test, a noxious stimulus, a high-intensity beam from a projector lamp bulb located below an unheated glass floor, is aimed at the plantar surface of the mid-hind paw. The latency in seconds to withdrawal or pain behavior is measured. Paw withdrawal caused by the stimulation is registered as a response. In the von Frey test, filaments of various thicknesses are applied against the central edge of the hind paw [14]. Increased paw withdrawal latency and filament thickness indirectly signify attenuated hyperalgesia. Furthermore, guarding behaviors such as a particular posture, limping and decreased weight bearing, licking and biting of an affected limb, and behavioral changes are used as indications of spontaneous pain [25]. In spite of the fact that behavioral pain assessments may not reflect the subjective pain experience in humans, these tests have been utilized in various studies. Behavioral assessments have been accepted as easily administrable and highly reproducible maneuvers having detailed and objective parameters that correlate with tumor-induced bone destruction [31].

To determine the mechanism of pain, several studies assessed pain-related factors that were upregulated during cancer pain and were decreased after electroacupuncture. Zhang et al. [20] used paw withdrawal latency (PWL) and spinal cord interleukin-1*β* (IL-1*β*) mRNA as outcome measures, thereby identifying the upregulation of spinal IL-1*β* mRNA that induced mechanical and thermal hyperalgesia. Another study [21] evaluated the hind paw withdrawal pressure threshold to assess mechanical hyperalgesia. Furthermore, preprodynorphin (PPD) mRNA and dynorphin were measured in the spinal cord in order to assess the role of dynorphin in cancer pain. Lee et al. [22] detected the upregulation of substance P during the cancer pain state, as well as increased *β*-endorphin production during electroacupuncture-induced analgesia. Kuai et al. [18] evaluated glial fibrillary acidic protein (GFAP). Factors upregulated in the cancer pain state, such as IL-1*β*, PPD mRNA, dynorphin, GFAP, and substance P were decreased after electroacupuncture treatment. This coincided with an improvement in pain-related behavior and suggested that these factors mediate the cancer pain mechanism.

Bisphosphonates are commonly used medications for metastatic bone disease [33]. These agents bind to the surface of the bone and directly cause apoptosis of osteoclasts, inhibit osteoclast-mediated bone resorption, and reduce tumor-associated osteolysis [6]. They inhibit tumor cell migration, invasion, adhesion, and cell proliferation, increase tumor-associated macrophages (TAMs), and increase the cytotoxicity of *γ* *δ*-T cells. Finally, bisphosphonates have shown synergistic antitumor activity with cytotoxic therapies [34, 35]. A recent therapeutic strategy is to target the bone microenvironment, because the complex interplay between cancer cells and the bone microenvironment induces bone metastasis [36].

From our results, outcome measures of pain-related behavior were used to evaluate the cancer pain and its mechanism including allodynia, hyperalgesia, and spontaneous pain. These behavioral responses to stimuli in animals with painful conditions are partially related to the subjective experience of pain in humans. In spite of feasibility and reproducibility of behavioral assessment measure of pain, parameters objectively and directly related to subjective pain experiences in humans describing tumor-induced bone destruction [32]. It is needed to be elucidated the study design to control factors that may exaggerate the effectiveness assessed using pain-related behavioral responses. Blinding maneuver and treatment assignments to prevent assessor bias were elucidated in three studies [18, 20, 21] while two studies did not described them [15, 22]. Studies of acupuncture for CIBP using bone microenvironment factors as outcome measures have not been conducted. Bone microenvironment factors such as osteoclasts, osteoblasts, endothelial cells, and hematopoietic progenitor cells may be considered to evaluate the mechanisms of CIBP. It will be required to establish new methods to evaluate pain that are directly related to subjective pain experiences in humans for future research.

This study has limitations stemming from the use of a single database (PubMed) and limited search strategies that returned a small number of studies. Very few studies have been reported on the effect of acupuncture on CIBP. In analyzing the papers in this study, it was difficult to follow a strictly standardized method of analysis because of limited access to complete data from the studies. Future researches are required to fully evaluate the effectiveness of acupuncture and the specific mechanism of CIBP in animal models.

## Figures and Tables

**Table 1:** Models of cancer pain.

Animal	Cell line	Tumor type	Strain	Sex	Injection site	Reference
Rat	Walker 256	Mammary gland carcinoma	Wistar	Female	Tibia	[15–18]
			Sprague-Dawley	Female	Tibia	[19]
	AT-3.1	Prostate carcinoma	Copenhagen	Female	Tibia	[20, 21]

Mouse	S-180	Sarcoma	BALB/c	Male	Femur	[22]

**Table 2:** Interventions and outcomes.

Reference	Intervention	Number of animals	Acupoint	Stimulation	Control	Number of animals	Days of EA treatment^a^	Outcome measure	Assessment days^a^	Outcome	Significance
[15]-1	EA	12	ST-36 BL-60	4 Hz 2.5 s/100 Hz 5 s, 1 mA, 30 min	Cancer only No intervention	12	Days 4~end of the study	Von Frey filament test (g)	10, 16, 22, 27	Increased Days 22, 27	*P* > 0.05

[15]-2	EA + Celebrex 5 mg/(kg∗g)	12	ST-36 BL-60	4 Hz 2.5 s/100 Hz 5 s, 1 mA, 30 min	Celebrex 5 mg/(kg∗g) EA (ST-36, 4 Hz 2.5 s/100 Hz 5 s, 1 mA, 30 min)	12 12	Days 4~end of the study	Von Frey filament test (g)	10, 16, 22, 27	Increased Days 10, 22, 27	*P* < 0.05

[20]-1	EA	7	GB-30	10 Hz, 2 mA, 0.4 ms, 30 min	Sham EA (GB-30, no stimulation)	7	Days 14–18	PWL^b^ (sec)	12, 15, 18	Increased Day 15 (9.18 ± 0.64 s) and Day 18 (9.19 ± 0.40 s)	*P* < 0.05

[20]-2	EA	7	GB-30	10 Hz, 2 mA, 0.4 ms, 30 min	Sham EA (GB-30, no stimulation)	7	Days 14–18	IL-1*β* (Spinal cord)	After behavioral test	Inhibited	*P* < 0.05

[21]-1	EA	7	GB-30	10 Hz, 2 mA, 0.4 ms, 30 min	Sham EA (GB-30, no stimulation)	7	Days 14–18	PWPT^c^ (g)	12, 14, 17	Increased	*P* < 0.05

[21]-2	EA	7	GB-30	10 Hz, 2 mA, 0.4 ms, 30 min	Sham EA (GB-30, no stimulation)	7	Days 14–18	PWL^b^ (sec)	12, 15, 18	Increased	*P* < 0.05

[21]-3	EA	7	GB-30	10 Hz, 2 mA, 0.4 ms, 30 min	Sham EA (GB-30, no stimulation)	7	Days 14–18	PPD mRNA (Spinal cord)	After behavioral test	Inhibited	*P* < 0.05

[21]-4	EA	7	GB-30	10 Hz, 2 mA, 0.4 ms, 30 min	Sham EA (GB-30, no stimulation)	7	Days 14–18	Dynorphin (Spinal cord)	After behavioral test	Inhibited	—

[18]-1	EA + morphine^d^	each 10	EX-B2	2 Hz/100 Hz, 30 min	Cancer only No intervention	10	Days 15–20	PWL^b^ (sec)	18, 20	Increased	*P* < 0.05

[18]-2	EA + morphine^d^	each 10	EX-B2	2 Hz/100 Hz, 30 min	Cancer only No intervention	10	Days 15–20	GFAP (Spinal cord)	After behavioral test	Inhibited	*P* < 0.01

[22]-1	EA	8	ST-36	2 Hz, 0.3 ms, <1 mA, 30 min	Cancer only No intervention	8	Days 1–9	Von Frey filament test (g)	1, 3, 5, 7, 9	Increased	*P* < 0.05

[22]-2	EA	8	ST-36	2 Hz, 0.3 ms, <1 mA, 30 min	Cancer only No intervention	8	Days 1–9	Cumulative lifting duration^e^ (sec)	3, 5, 7, 9	Increased	*P* < 0.05

[22]-3	EA	8	ST-36	2 Hz, 0.3 ms, <1 mA, 30 min	Cancer only No intervention	8	Days 1–9	Substance P (Spinal cord)	After behavioral test	Decreased	*P* < 0.05

[22]-4	EA	8	ST-36	2 Hz, 0.3 ms, <1 mA, 30 min	Cancer only No intervention	8	Days 1–9	*β*-endorphin (Brain)	After behavioral test	Increased (4.355 ± 0.2972 ng/mL)	*P* < 0.05

EA, electroacupuncture; ST-36, Zusanli; BL-60, Kunlun; GB-30, Huantiao; EX-B2, Jiaji; IL-, interleukin-; PPD, preprodynorphin; GFAP, glial fibrillary acidic protein.

^a^Days after inoculation.

^b^PWL, Paw withdrawal latency (thermal sensitivity to radiant heat).

^c^PWPT, Paw withdrawal pressure threshold measured with a Paw Pressure Analgesia Instrument (UgoBasile, Italy).

^d^Electric current intensity of EA, 2 mA, 1 mA; dose of morphine, 0 mg/(kg∗d), 2.5 mg/(kg∗d), 5 mg/(kg∗d).

^e^Cumulative lifting duration: After approximately 1 h acclimatizationin a clear plastic chamber with wire grid floors at room temperature, the cumulative duration of hind paw-lifting of each mouse was analyzed for 10 min.

**Table 3:** Outcome measures.

Reference	Behavior			Macroscopicfeatures	Histological and biochemical measures (site)
	Mechanical stimulus evoked	Heat stimulus evoked	Movement related		
[15]	Hind paw withdrawal (von Frey filaments)			Bone surface Bone destruction (X-ray)	

[20]		Paw withdrawal latency (thermal sensitivity to radiant heat)			IL-1*β* (spinal cord)

[21]	Hind paw withdrawal pressure threshold (PWPT)	Paw withdrawal latency (thermal sensitivity to radiant heat)			PPD mRNA, Dynorphin (spinal cord)

[18]		Paw withdrawal latency (thermal sensitivity to radiant heat)		Bone surface Bone destruction (X-ray)	GFAP (spinal cord)

[22]	Hind paw withdrawal (von Frey filaments)	Cumulative lifting duration	Clear lifting and flinching	Tumor size and volume (MRI scanning)	Substance P (spinal cord) *β*-endorphin (brain)

IL-, interleukin-; PPD, preprodynorphin; GFAP, glial fibrillary acidic protein.
